# Treatment Response and Drug Resistance Profiling of Genotype 6 of Hepatitis C Virus in HCV/HIV Co-Infected Patients: A Pilot Study from INDIA

**DOI:** 10.3390/v14050944

**Published:** 2022-04-30

**Authors:** Ekta Gupta, Jasmine Samal, Amit Pandey, Gaurav Singh, Hajra A. S. Gupta, Reshu Agarwal, Manoj Kumar Sharma

**Affiliations:** 1 Department of Clinical Virology, Institute of Liver and Biliary Sciences, New Delhi 110070, India; samaljasmine@gmail.com (J.S.); amitpdy23@gmail.com (A.P.); gaurav_singh985@yahoo.com (G.S.); agarwalreshu286@gmail.com (R.A.); 2 Department of Innate Immunity, ICMR-National Institute for Research in Reproductive and Child Health, Mumbai 400012, India; hajra.gupta11@gmail.com; 3 Department of Hepatology, Institute of Liver and Biliary Sciences, New Delhi 110070, India; manojkumardm@gmail.com

**Keywords:** HCV GT6, occurrence, chronic hepatitis C, HCV/HIV, co-infection, phylogenetic analysis, SVR, RAS

## Abstract

Hepatitis C Virus (HCV) genotype (GT) 6 demonstrates maximum genomic diversity out of all the known genotypes of HCV, attributable to its inherent intra-genotype and inter-genotype recombination property. This is the most common genotype seen in HCV/HIV co-infected cases. HIV/HCV co-infection is linked with increased genetic diversity in HCV structural genes. The detailed information on the distribution of HCV GT6, its subtypes, and resistance to currently available antiviral drugs is limited in the Indian subcontinent. Therefore, in this single-center retrospective cross-sectional study, we aimed to map the occurrence of HCV GT6, its subtypes and resistance-associated substitution (RAS), and its correlation with antiviral treatment response in HCV-infected patients. From a cohort of 2052 HCV-infected patients, the overall prevalence of GT6 was 2.5% (*n* = 53), with a maximum of 81.1% (*n* = 43) seen in HCV/HIV co-infected patients. Nine different subtypes, 6a, 6b, 6f, 6i, 6n, 6u, 6v, 6w, and 6xa, were detected in the Indian population for the first time, with a predominance of 6xa (41.5%), a rare subtype, followed by 6n (39.6%). The phylogenetic analysis by the neighbor-joining method revealed three prominent viral clades, 6v, 6n, and 6xa–6u. The baseline (before treatment initiation) plasma samples of all GT6-infected patients were retrieved from −80 °C and a part of the NS5a and NS5b region of the viral genome was analyzed for the presence of RAS. No RASs were seen in the NS5b region, while in two patients (3.7%) RASs were seen at baseline in the NS5a region of the virus. Sustained viral response (SVR) was attained in 81% (*n* = 43) of patients. No difference in GT6 subtype distribution or occurrence of RAS was seen between mono-infected HCV and HIV/HCV co-infected cases. Our study revealed that RAS at baseline did not influence the attainment of SVR and the currently available antiviral therapy is effective against GT6 mono-infected and HIV/HCV co-infected patients.

## 1. Introduction

Hepatitis C Virus (HCV), an enveloped, positive-sense single-stranded RNA virus, is linked to liver-related morbidity and mortality. It is estimated that about 3% of the world’s population remains chronically infected with HCV, accounting for 170 million individuals infected with HCV worldwide. Infection with HCV often evolves into chronic hepatitis C (CHC), potentially advancing to liver cirrhosis, fibrosis, and hepatocellular carcinoma. The disease inflicts immense health and economic burden on endemic countries owing to hepatic and extrahepatic manifestations associated with it. HCV exhibits a high rate of genetic variations within the genome, resulting in seven different genotypes (GT1, 2, 3, 4, 5, 6, and 7) with several confirmed subtypes to date [1]. Enhanced incidence of HCV is reported from high-risk populations, including HIV-infected groups [2], intravenous drug users, and individuals with sexually transmitted diseases [3]; this necessitates studies evaluating the genotype prevalence and existence of clinically relevant natural resistance-associated substitutions (RAS) in them. Host immune responses influence the genetic diversity of HCV, especially in HIV co-infection, and genetic variations in the structural genes of the HCV virus have been observed [4]. Treatment response and disease management also differ in the HCV genotypes [5]. HCV GT1 and 3 are the predominant genotypes globally, with the predominance of GT3 in the Indian population [6].

Previous studies have reported the prevalence of HCV GT6 among the Indian population, particularly from the north-eastern region [7,8]. A retrospective study by Panyala et al. demonstrated the association of temporal changes with the prevalence of HCV genotypes (Genotype 1 being predominant) over seven years in a tertiary health care setting [6]. Intriguingly, the prevalence of HCV GT6 is becoming apparent in high-risk groups with limited information on real-life treatment responses. Epidemiological studies have shown that HCV GT6 is most prevalent in south-east Asia and accounts for approximately 5% of all HCV infections [9]. A study by Jia et al. identified a new HCV GT6 subtype, 6xj, and baseline RAS associated with it. They demonstrated that 28V and 32L substitutions did not affect attainment of sustained biological responses [10]. In addition, literature regarding genetic diversity within HCV GT6, its epidemiological manifestations, and prevalence of RASs is still insufficient. It is believed that repeated viral exposure during intravenous drug use and sexual contact leads to the circulation of more than one subtype of HCV, promoting viral recombination and increasing the risk of the emergence of resistant HCV strains [11]. Thus, in this pilot study, we estimated the occurrence of HCV GT6 and its subtype in the Indian population and identified naturally occurring baseline RASs (BL-RAS) for GT6.

## 2. Materials and Methods

This is a retrospective study conducted at the Institute of Liver and Biliary Sciences, New Delhi, from January 2011 to July 2021, where records of clinician-initiated testing for HCV genotyping were available in the laboratory database. The details of patient history, diagnostic test results, CD4 cell counts, and clinician-recommended treatment regime were sorted for data analysis. Out of all the patients infected with HCV, the study included patients infected with HCV GT6 (as per the inclusion criteria). The HCV GT6 positive patients with a history of an HIV co-infection were also included in the study. The distribution of HCV GT6 subtypes was also calculated to gather a geographical estimate of the variant viral population. The plasma samples of the recruited patients (before therapeutic intervention) were thawed from a deep freezer, and were used for RNA extraction and sequenced by direct PCR-based sequencing for the NS5a and NS5b regions of HCV as described in our previous study. For identification of GT6 RAS in the NS5a region, the following primers were used: Fwd primer (6103 position)-5′GRGGBAAYCAYGTNTCHCCHAC3′, Rev primer (6867 position)-5′TGDGAKGGRTCDGTBARCATKGA3′. The NS5b region of HCV GT6 was amplified using the following primers: Fwd primer (7980 position)-5′TGGRAGGACYTGYTRGARGAC3′, Rev primer (8661 position)-5′GRGGNGCNGARTAYCTGGTC3′. The FASTA sequences of the NS5b region of the viral variants were subjected to phylogenetic analysis. The phylogenetic tree was constructed by the maximum likelihood method using the Kimura-2 substitution model (K2 + G + I) to further enhance tree accuracy, followed by bootstrapping with 100 replicates in MEGA software (version 6.06). The patients were prescribed a combination drug regime of Sofosbuvir (SOF) and Daclatasvir (DCV) with/without Ribavirin (RBV) as per the National guidelines. Sustained Virological Response (SVR) is defined as undetectable HCV RNA 12 weeks after treatment completion (as per WHO guidelines). SVR12 is considered equivalent to the cure of HCV infection. The study was approved by the Institutional Ethics Board (IEC/2020/79/MA03) and conducted as per the principles of the Declaration of Helsinki. The study was performed on de-identified, anonymous, archived clinical plasma samples. Hence, the requirement for individual patient consent forms was waived.

## 3. Results

### 3.1. Distribution of HCV GT6 and Its Subtypes

**Figure 1:**
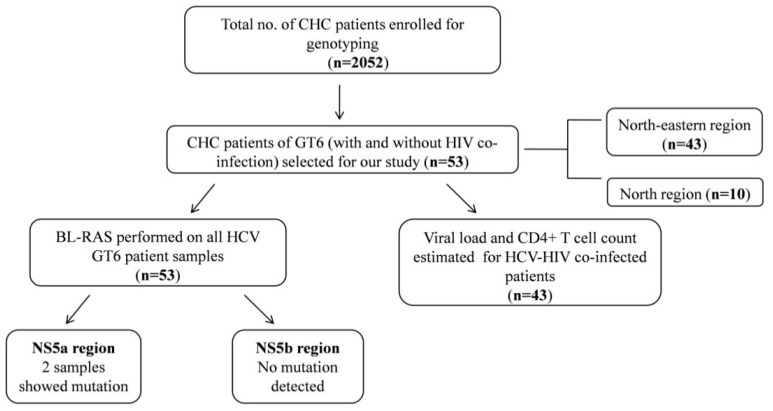
A schematic representation of workflow of this study.

Genotype information of 2052 HCV positive patient samples was available from the institutional database. A schematic diagram of our workflow has been depicted in Figure 1. Patients had a mean age of 39 (±11.5 SD) years, the majority (69.8%) being male. Median HCV RNA levels (IU/mL) in log_10_ were: 6.39 (IQR: 5.67–6.79) and 19 (35.8%) patients were cirrhotic as per the records.

**Figure 2:**
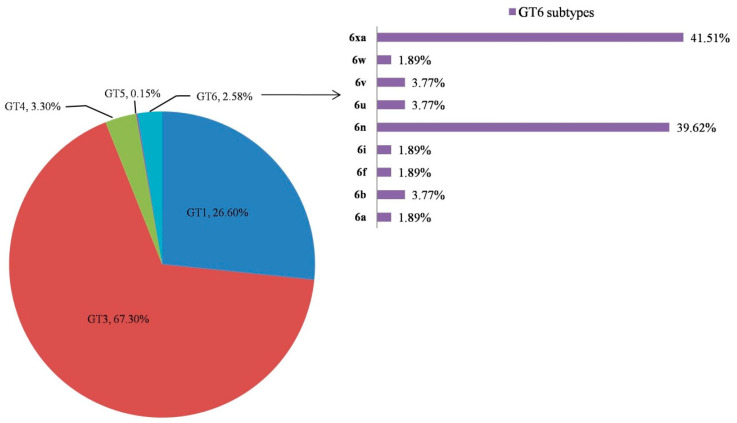
A pie chart indicating the distribution of different genotypes of HCV and subtypes of HCV GT6 in our enrolled population.

The GT distribution in 2052 enrolled patients: GT3 in 1382 (67.3%), GT1 in 546 (26.6%), GT4 in 68 (3.3%), GT6 in 53 (2.5%) and GT5 in 3 (0.1%) (Figure 2). In the selected cohort of GT6 (*n* = 53), 9 subtypes, including common (6a and 6n) and 7 rare subtypes, were seen, viz. 6b, 6f, 6i, 6u, 6v, 6w and 6xa. Among these, there was a predominance of 6xa (41.5%) followed by 6n (39.6%) (Figure 2). 

#### Phylogenetic Analysis of HCV GT6 Subtypes

**Figure 3:**
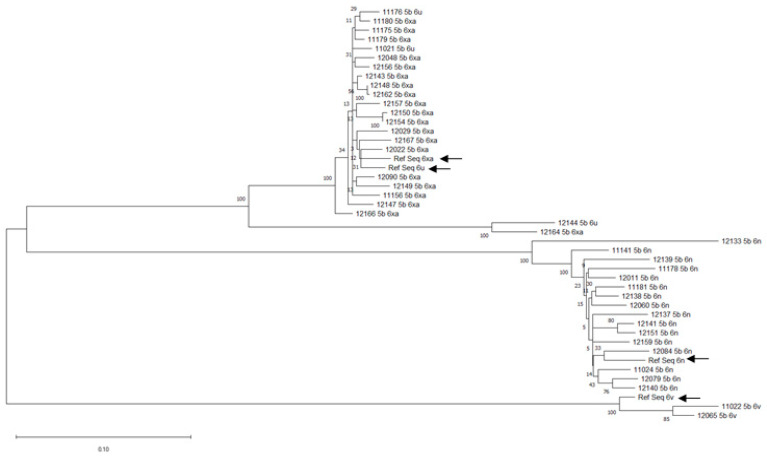
A phylogenetic tree of HCV GT6 subtypes. The tree generated by maximum likelihood shows the emergence of three viral clades, 6xa–6u, 6n, and 6v. The ingroups are identified as 6xa, 6u, and 6n, while 6v is the outgroup. The reference sequences for each subtype are indicated by black arrows.

The NS5B sequences of the viral subtypes were aligned by multiple sequence alignment (gapped) using the MUSCLE algorithm in MEGA. Four reference sequences for each subtype—6xa (KP323812.1), 6n (AB291038.1), 6v (KP323811.1), and 6u (KX940866.1) were obtained from the NCBI nucleotide database and included in the alignment to validate the tree. Based on the alignment generated by MUSCLE, the best-fit substitution model selection test was performed. The results were generated by the maximum likelihood method and confirmed the choice of Kimura 2 +G + I as the best-fit substitution model with the lowest AICc and BIC scores in the list of twenty-four fits obtained as hits. Subsequently, the phylogenetic tree yielded an ingroup of subtypes 6n, 6xa, 6u, and an outgroup of subtype 6v with a node score of 100. These two groups could be attributed to the status of parental subtypes, both arising from a common ancestral variant (Figure 3). The ingroup subtypes (giving 6n, 6xa, and 6u) diverge to evolve into two offspring variants, 6n and 6xa, 6u, each with an independent node score of 100. The reference sequences for each subtype appeared in their respective viral subtype clades (Figure 3).

### 3.2. Correlation of HCV GT6 Subtypes with Viral Load and CD4+ T Cell Count

Notably, most of the patients with HCV GT6 were co-infected with HIV (*n* = 43, 81.1%) with a median CD4+ T cell count of 413 (IQR 223.7–546.7) cells/mm^3^, and all the HCV-HIV co-infected patients belonged to the Northeast states of India. There was a significant difference between HCV GT6 subtypes and their corresponding viral load (*p* = 0.0058), with the highest viral load of 1.48 × 10^7^ IU/mL and 10^7^ IU/mL for subtypes 6v and 6f respectively, and the lowest viral load of 1.5 × 10^3^ IU/mL for subtype 6a. There was, however, no significant difference among HCV GT6 subtypes towards attainment of treatment response (*p* = 0.755). In patients co-infected with HIV, the count of CD4+ T cells/mm^3^ showed a significant variation between the subtypes harbored by the patient, with the highest CD4+ T cell count of 565 cells/mm^3^ and 549 cells/mm^3^ for subtype 6f and 6a respectively, and lowest CD4+ T cell count of 106 cells/mm^3^ for subtype 6i (*p* = 0.0355) (Table 1).

### 3.3. Occurrence of BL-RAS and Its Association with the Treatment Response

We examined the sequences of HCV GT6 for the presence of RAS as per our previously published protocol [12]. None of the viral variants carried any naturally occurring RASs in their NS5b region, whereas two samples carried mutation at the NS5a region (one patient with T93A and another one with T93S). 

The patients had been prescribed a combination drug regime of Sofosbuvir (SOF), Daclatasvir (DCV), and Ribavirin (RBV) with combinations of SOF-DCV or SOF-DCV-RBV as per the National guidelines [13]. We defined a successfully completed regimen when SVR was attained i.e., HCV RNA levels were below the limit of quantification at 12 weeks post-treatment. Treatment failure in patients was considered when detectable HCV viral load was present 12 weeks post-treatment. Attainment of SVR inversely correlates with viral load (*p* = 0.015, Table 1). In our study, SVR was attained in 81% (*n* = 43) of CHC patients with direct-acting antiviral (DAA) therapy. Although the existence of RAS (NS5a region) at baseline did not influence the attainment of SVR, we need large sample size studies to draw firm confirmations on the impact of BL-RAS on treatment response in HCV GT6 infected patients.

## 4. Discussion

HCV infection is a public health concern in developing countries, especially in Asia and Africa. Emerging genotypes and subtypes of HCV have contributed to the severity of the disease with the generation of viral variants and drug-resistant mutants. Globally, the burden of HCV infection is primarily due to HCV 1 and 3 genotypes. Despite geographical restrictions, HCV GT6 exhibits the highest genetic diversity, with 23 known subtypes identified in the last decade. In Asian countries, infection from HCV GT6 is most prevalent in Thailand, Myanmar, China, and Hong Kong [14]. HCV/HIV co-infection is often associated with increased genetic diversity in the HCV structural genes, probably due to immune pressure or viral persistence [4,15]. GT6 is the most prevalent genotype in the HCV/HIV co-infected cohort. Current literature available on epidemiology and treatment response of HCV GT6 is limited and warrants further investigation. The present study, which is the first of its kind from this part of the world, highlights the treatment response of HCV GT6 to standard DAAs and investigates the different subtypes in this genotype. In our study, GT6 was seen in 53 patients (2.5%), slightly higher than previously reported in our country [6], probably as our Institute is a dedicated tertiary care liver institute, hence most difficult to treat cases are referred to our Institute from different parts of our country.

A clear predominance of HCV GT6 was seen in our study among the north-eastern population, which concurs with a previous report showing HCV GT6 as the most prevalent genotype among eastern and north-eastern parts of India [7]. A study by Barman et al. from the north-eastern part of India, demonstrated that approx. 30% of the HCV sequences, isolated from CHC patients, belong to GT6, second to GT3 [16]. A previous study from our group has reported HCV GT6 as the dominant GT in HCV/HIV co-infected cohort, [12]. Detailed information on HCV genotyping and sub-genotyping and its geographical mapping could contribute immensely to the effective elimination of HCV from our country and faster achievement of the global goal of HCV elimination by 2030.

Understanding the treatment response to conventional drugs for all existing HCV genotypes in our population is important if we wish to extend our National Viral Hepatitis Control Program to reach the sub-district level of control (National Viral Hepatitis Control Program-Operational Guidelines, 2018 by the Ministry of Health and Family Welfare, Government of India). This work is the first attempt to enumerate the types and numbers of HCV GT6 subtypes in the Indian population. In the selected cohort, nine subtypes were discovered, viz. 6b, 6f, 6i, 6u, 6v, 6w and 6xa. Among these, there was a predominance of 6xa (41.5%) followed by 6n (39.6%); a predominance of 6n was also reported in Myanmar [17], mainland China [18], and Thailand [19]. The prevalence of the 6xa subtype was almost 50% in the study cohort, which is one of the rarest HCV GT6 subtypes. Patients with HCV/HIV co-infection showed significant differences between CD4+ T cell numbers and the viral subtype they carried. The prevalence of HIV/HCV co-infection is significantly high among injection drug users (IDU). Previous studies conducted in north-eastern India showed that more than 90% of HIV-infected people (among IDU) had HCV co-infection, emphasizing the need to screen HIV-infected drug users for HCV infection and initiate timely and appropriate treatment [20,21]. Though our results are promising, they need to be validated in a large prospective cohort before drawing any general conclusions.

From the phylogenetic tree, three viral clades emerge, viz. 6v, 6n, and 6xa–6u. Thus, three individual subtype populations can be identified. The emergence of the three clades from two distinct groups (ingroup and outgroup) makes certain one key fact: the divergent evolution of 6v from 6n, 6xa, and 6u. The 6n subtype is predominant in most countries; therefore, its separation from 6xa and 6u in the tree appears to be an evolutionary mechanism to increase its infectivity. The spontaneous emergence of 6u from parent roots of 6xa may indicate either a random mutation event or the initiation of another dominant viral subtype, as is evident from the high node scores generated in the maximum likelihood. More data on the prevalence of 6u in larger cohorts from longitudinal studies will help validate this data’s hypothesis. An exciting finding was the divergence of the 6v subtype, desperately attempting to increase its prevalence among the pathogenic variants by incorporating more mutations. Despite significant advances in anti-HCV treatment and therapy (DAAs), 100% SVR is still not possible and the most important cause attributable to treatment failure, after non-adherence, is the existence of RAS in the viral genome [22].

In our study, none of the viral variants carried any naturally occurring RASs in their NS5b region; whereas two samples carried one substitution each at the NS5a region (one patient with T93A change and another with T93S change). We further evaluated the impact of RAS on treatment response. Our findings show that SVR was attained in 81% (*n* = 43) of CHC patients with DAA treatment. We found the presence of minimum RAS in this genotype and the RAS responsive to currently existing treatment modalities. In addition, Michaluk et al. demonstrated that SVR attained in HCV GT4 mono-infected patients did not differ significantly from HCV/HIV co-infected patients, underlining the safety and effectiveness of current DAA therapy for HCV/HIV co-infected patients [23]. A study by Flower et al. demonstrated no contribution of RAS to treatment outcomes in a cohort in Vietnam where HCV GT6 was predominant [24]. Although the existence of RAS (NS5a region) at the baseline did not influence the attainment of SVR in our analysis, we need large sample size studies to draw firm confirmations on the impact of BL-RAS on treatment response in HCV GT6 infected patients.

Since studies demonstrate that IDUs are at an increased risk for HCV/HIV co-infection, the link between HCV prevalence and socio-behavioral aspects warrants further investigation. This would facilitate large-scale screening for HCV infection among the susceptible population, especially among HIV-infected drug users, and would ensure timely diagnosis and treatment.

With the study’s highlight being the discovery of the prevalence of HCV GT6, a few shortcomings limit the study’s extrapolation:(1) A limited cohort size was selected for the study, which may have skewed accurate numbers if it had been a larger population. (2) The schedule of therapeutic intervention was uneven in the group; therefore, it may have either overestimated or underestimated the numbers of CD4+ T cells and, hence, the effect and outcome of antiretroviral therapy. (3) The correlation between HCV GT6 and the spectra of liver disease is not studied. (4) Since ours is a tertiary healthcare center, the overall data is not a true reflection of the distribution of HCV GT6 in the Indian population. Despite these limitations, our study provides an up-to-date report on the situation of the distribution of HCV genotypes, their subtypes, and the impact of BL-RAS on treatment outcomes.

## 5. Conclusions

Our study contributes toward understanding the prevalence of HCV genotypes, particularly GT6 among CHC patients. This retrospective study testifies to the increase in the incidence of HCV GT6 infection in the Indian population since 2015, and the prevalence and distribution of HCV GT6 subtypes, 6xa and 6n, as being predominant. It corroborates the notion of a higher prevalence of HCV GT6 among the north-eastern population in India, especially among HIV/HCV co-infected individuals. Large-scale prospective studies, assessing epidemiology and rates of SVR attained among HCV GT6 mono-infected and HCV/HIV co-infected patients in the Indian population, are imperative. Thus, our study reinforces the need for national surveys and statistics to determine the burden of HCV GT6 as a risk factor for the development of severe liver disease.

## Figures and Tables

**Table 1:** A table showing baseline demographics, CD4+ T cell count, and viral load of the patients enrolled in our study.

Variables	Count	*p*-Value	Significance
**Demographics**			
Age	44 ± 12.40		
Gender	Male 37/53 (69.8%)		
	Female 16/53 (30.2%)		
**Patient Characteristics**			
Total subjects	53/2052 (2.5%)		
HCV GT6 co-infection with HIV	43/53 (81.1%)		
HCV GT6 infection alone	10/53 (18.9%)		
**HCV GT6 subtypes distribution**			
6a, 6f, 6i, 6w	1/53 (1.9%)		
6b, 6u, 6v	2/53 (3.8%)		
6n	21/53 (39.6%)		
6xa	22/53 (41.5%)		
**Gender**			
Viral Load		0.263	NS
Subtype		0.077	NS
Treatment Outcome		0.123	NS
**HCV GT6 subtypes**			
Viral Load		0.006	S
CD4+ T Cell Count		0.036	S
Treatment Outcome		0.755	NS
**Treatment Outcome**			
Viral Load		0.171	NS
CD4+ T Cell Count		0.652	NS
Choice of Therapy		0.066	NS
**Sustained Virological Response (SVR)**			
Viral Load		0.015	S
CD4+ T Cell Count		0.197	NS
Choice of Therapy		0.182	NS

Abbreviations: NS-Not significant, S-Significant.

## Data Availability

The data for sequencing is available at GenBank (MT032265-MT032305).
